# The role of renin angiotensin system inhibition in kidney repair

**DOI:** 10.1186/1755-1536-3-7

**Published:** 2010-05-04

**Authors:** Irene M van der Meer, Paolo Cravedi, Giuseppe Remuzzi

**Affiliations:** 1 Mario Negri Institute for Pharmacological Research, Bergamo, Italy; 2 Unit of Nephrology, Azienda Ospedaliera Ospedali Riuniti, Bergamo, Italy; 3 Department of Internal Medicine, Division of Nephrology, University Hospital Maastricht, Maastricht, The Netherlands

## Abstract

Chronic kidney diseases share common pathogenic mechanisms that, independently from the initial injury, lead to glomerular hyperfiltration, proteinuria, and progressive renal scarring and function loss. Inhibition of the renin angiotensin system (RAS) has been consistently found to reduce or halt the progressive deterioration of renal function through reduction of blood pressure and proteinuria, the two main determinants of renal function decline. In few instances, RAS inhibition may even promote amelioration of the glomerular filtration rate. Animal data suggest that chronic therapy with angiotensin-converting enzyme inhibitors or angiotensin II receptor type I blockers promotes regression of glomerulosclerosis, even in later phases of the disease. In humans, studies investigating the effect of angiotensin II inhibition on renal structural changes have shown inconsistent results, possibly due to small numbers and/or short duration of follow-up. Whether regression of glomerulosclerosis relies on a direct regenerative effect of RAS inhibition or on spontaneous kidney self-repair after the injury has been removed is still unknown. Improved understanding of mechanisms that promote renal regeneration may help in designing specific therapies to prevent the development of end-stage renal disease. This is a desirable goal, considering the economic burden of chronic kidney diseases and their effect on morbidity and mortality.

## Introduction

Chronic kidney disease (CKD) represents a major health problem worldwide. It has been estimated that approximately 830,000 deaths every year are linked to renal diseases, but the scale of the problem is probably largely underestimated [1] Costs for renal replacement therapies cannot be afforded on a population basis by most developing countries, and estimates predict that economic expenses for these treatments are becoming very problematic for wealthier nations too. Therefore, identifying mechanisms that sustain renal disease progression and those that allow recovery of renal function and structural integrity after injury will be crucial to develop hypothesis-driven therapies able to promote remission or even regression of CKD. This will be of even higher importance for global morbidity and mortality because renal impairment also represents a major risk factor for cardiovascular disease [1].

Independently from the initial insult, chronic nephropathies seem to share common pathogenic mechanisms leading to progressive renal function loss and fibrosis [2]. Targeting blood pressure (BP) and proteinuria can reduce the rate of kidney function decline and prevent or delay the need for renal replacement therapy in many patients. The cornerstone of current treatment is inhibition of the renin angiotensin system (RAS), which has been consistently described to improve renal function with concomitant regression of kidney structural changes in animal models. Similar effects have been reported in selected patients, providing evidence that the kidney has some regenerative capacity that might be boosted by specific and targeted treatment [3,4]. In this paper, we first review the evidence both from experimental studies and from clinical studies in humans that RAS inhibition can induce renal structural and functional changes. We then provide an extensive summary of the possible direct or indirect mechanisms by which inhibition of the RAS contributes to kidney repair.

## Pathophysiology of renal disease progression

Experimental and clinical research has clearly documented that, independently from the initial injury, renal disease progression is sustained by common mechanisms that, starting from nephron loss, lead to compensatory glomerular hemodynamic changes. In the experimental model of renal mass reduction by five-sixths nephrectomy, resembling advanced phases of CKD, the remnant glomeruli undergo hypertrophy, and the tone of afferent arterioles drops more than that of efferent arterioles [5]. This increases glomerular capillary hydraulic pressure, leading to more filtrate formed per nephron (hyperfiltration). These changes initially minimize the functional consequences of nephron loss, but ultimately are detrimental, causing progressive injury of the remaining intact nephrons. Increased intraglomerular capillary pressure and perfusion pressure result in mechanical damage to the three major cell types in the glomerulus (the podocytes, endothelial cells and mesangial cells) leading to impaired selectivity of the glomerular capillary wall and excessive protein ultrafiltration [5].

A key player in these glomerular hemodynamic changes, crucial to progressive renal injury, is angiotensin II [6]. Indeed, glomerular capillary hypertension is often maintained by angiotensin-dependent mechanisms via increased systemic BP and vasoconstriction of the efferent arterioles. Beyond causing glomerular hypertension, angiotensin II has been suggested to promote progressive renal damage directly through a variety of mechanisms, including increased extracellular matrix (ECM) deposition, immune activation and induction of growth factor release [7]. Moreover, angiotensin II alters the size-selective properties of the glomerular capillary barrier, which further increases protein filtration into the urinary space [8].

### Consequences of glomerular permeability dysfunction and proteinuria

Podocyte injury secondary to glomerular hypertension and the direct effects of angiotensin II leads to increased protein ultrafiltration in the urinary space. When proteinuria is highly selective, that is, when albumin represents its major component, tubulointerstitial damage and renal function loss is rather infrequent. Conversely, when larger proteins also pass through the glomerular barrier into the urinary space, tubulointerstitial damage takes place and renal function progressively declines [9]. Consistently, longitudinal studies in diabetes mellitus type 1 (T1DM) and type 2 (T2DM) clearly show that the glomerular filtration rate (GFR) in general starts to decline only with the appearance of macroalbuminuria, that is, when proteins larger than albumin appear in the urinary space [10-12].

Protein overload in the tubules induces tubular cells to release cytokines, chemokines, growth factors and vasoactive substances, which leads to abnormal interstitial accumulation of inflammatory cells, ECM collagen, fibronectin and other components that are responsible for interstitial fibrosis [13]. Notably, glomerular permeability dysfunction results in the passage of complement factors into Bowman's space and the tubular lumen. Moreover, tubular cells themselves synthesize complement factors under stress conditions, causing an abnormally high exposure of epithelial cells to these reactive proteins [14,15]. Consistently, abnormal C3 and C5b-9 staining in proximal tubular cells and along the brush border is a well-known feature both in human chronic proteinuric diseases and experimental models, and complement activation is now known to be a powerful mechanism underlying tubular and interstitial injury by exerting cytotoxic, proinflammatory and fibrogenic effects [16].

Filtered oxidized lipoproteins can promote lipid accumulation in glomerular, tubular and interstitial cells, which in turn promotes progressive renal function loss [17]. Moreover, tissue injury induced by proteinuria promotes the generation of reactive oxygen species and an endoplasmic reticulum stress response by renal cells [18]. This leads to the oxidative modification of membrane lipids, proteins and DNA, thereby initiating cell-death responses that result in tissue inflammation and local recruitment of macrophages and lymphocytes, and so further fuel the inflammatory process [18,19]. This altered interstitial milieu promotes epithelial-mesenchymal transition (EMT), a process by which differentiated epithelial cells undergo a phenotypic conversion into matrix-producing fibroblasts and myofibroblasts. This process is increasingly being recognized as an integral part of tissue fibrogenesis after injury, and could be an adaptive response of epithelial cells to an unfavorable microenvironment [20]. Recent studies showed that not only tubular epithelial cells, but also endothelial cells and glomerular podocytes may undergo mesenchymal transition after injury, leading to functional impairment and glomerulosclerosis [20,21].

The aforementioned mechanisms sustain progressive renal function loss and scarring, which, if left untreated, inexorably leads to end-stage renal failure (ESRF). Thanks to compensatory adaptations by the high number of unimpaired nephrons, this does not immediately translate into changes in the renal function parameters most commonly used in the clinical practice, such as serum creatinine and urea. Beyond a certain level of injury, however, compensatory adaptations no longer keep pace with nephron loss, and glomerular filtration rate declines.

## Prevention of progressive renal function decline with RAS inhibition: results from clinical trials

Given the important role of angiotensin II in inducing and sustaining glomerular hypertension and proteinuria and its deleterious consequences, clinical studies have focused on the renoprotective effects of angiotensin II inhibition.

The Angiotensin-Converting Enzyme Inhibition in Progressive Renal Insufficiency (AIPRI) study showed that angiotensin-converting enzyme (ACE) inhibitor therapy reduced the risk of serum creatinine doubling in 583 mainly non-diabetic patients with chronic proteinuric nephropathies [22]. However, a much more effective BP reduction on ACE inhibitors did not allow for a conclusion about whether this effect was specific for the ACE inhibitor therapy itself or merely reflected better control of arterial hypertension. Stronger evidence of the renoprotective effect of ACE inhibitors were provided by the Ramipril Efficacy in Nephropathy (REIN) study, showing that with comparable BP control, treatment with the ACE inhibitor ramipril reduced the rate of GFR decline and progression to ESRF in patients with non-diabetic proteinuric nephropathies compared with placebo. Patients with higher levels of baseline proteinuria benefited the most [23,24]. In a subsequent meta-analysis of 1860 non-diabetic patients, it was again shown that ACE inhibition significantly reduced the risk of creatinine doubling or kidney failure [25]. Importantly, the risk reduction was limited to patients with ≥ 500 mg urinary protein excretion per day, further supporting the pathogenic role of proteinuria in promoting renal damage.

Although the responsiveness to antiproteinuric therapy may vary between the different non-diabetic glomerulopathies, the aforementioned studies were not designed to detect such differences. However, efficacy of angiotensin II inhibition has been reported for diverse proteinuric diseases including IgA nephropathy [26], membranous nephropathy [27], and HIV-associated nephropathy [28]. For primary focal and segmental glomerular sclerosis, data regarding the efficacy of angiotensin II inhibition are scarce [29].

Of note, current evidence supports the benefit of RAS inhibition even in the advanced phases of renal failure. A *post hoc *analysis of the REIN study showed that the efficacy of ACE inhibitor therapy over placebo in prevention of ESRF was consistent across all levels of baseline GFR [30]. Even more compelling evidence of the beneficial effect of ACE inhibitor therapy was provided by Hou *et al*., who randomly assigned 224 non-diabetic patients with an estimated GFR < 30 mL/min to benazepril or placebo [31]. After 3.4 years of follow-up, the number of patients progressing to ESRF was 40% lower in the group of patients on benazepril compared with the placebo group (*P *= 0.02), despite similar BP control. Thus, RAS inhibitors should be continued up to the very advanced phases of renal failure. However, when GFR declines to < 15 mL/min, RAS inhibitor discontinuation may be considered, in order to increase GFR and retard the need for renal replacement therapy[32].

In (incipient) diabetic nephropathy, ACE inhibitors and angiotensin II receptor type I blockers (ARBs) also exert their renoprotective effects. In hypertensive T1DM or T2DM patients with microalbuminuria or overt nephropathy, both ACE inhibitors and ARBs protected against the progression of renal disease independent of BP reduction [33-37]. Moreover, it was shown that in normoalbuminuric hypertensive T2DM patients, trandolapril halved the risk of developing microalbuminuria compared with conventional therapy [38]. This is of major importance because microalbuminuria strongly predicts diabetic nephropathy, and T1DM or T2DM patients with microalbuminuria have a 21-fold and 9-fold increased risk, respectively, of developing diabetic nephropathy compared with those without microalbuminuria [39,40].

### ACE inhibitors versus ARBs: is there any difference?

Only few studies have addressed the question of whether ACE inhibitors are better than ARBs or *vice versa*. In the Renoprotection of Optimal Antiproteinuric Doses (ROAD) study, non-diabetic proteinuric patients were randomized to either benazepril or losartan in the conventional dose or in a dose that was titrated upwards until the maximum antiproteinuric efficacy was reached [41]. This study clearly demonstrated that the titrated therapy reduced the incidence of the combined endpoint of serum creatinine doubling, ESRF or death after a median follow-up of 3.7 years. Importantly, the effects of the strategy did not depend on the type of drug; the results were not different between benazepril and losartan. Another study, comprising type 2 diabetic patients with incipient or overt nephropathy, showed that there was no significant difference in the effects of enalapril and telmisartan on measured GFR decline after 5 years of treatment, although the decline in GFR tended to be lower in patients on enalapril [42]. Other studies in hypertensive type 2 diabetics with early nephropathy comparing ACE inhibitors and ARBs have also failed to show significant differences in the effects of these two drug classes on BP and urinary albumin excretion [43,44]. These findings are particularly important for renal disease prevention and treatment in low-income countries, as ACE inhibitors are much less costly than ARBs [45].

### Double RAS inhibition to maximize proteinuria reduction

Double RAS inhibition may have a superior antiproteinuric effect to single inhibition with either agent. In patients with persisting proteinuria, the addition of an ARB to an ACE inhibitor counteracts the effects of angiotensin II that is produced via ACE-independent pathways, whereas the addition of an ACE inhibitor to an ARB limits compensatory angiotensin II production. Direct comparisons of combined RAS blockade versus single blockade are scarce, and the only moderately large study reported to date has recently been retracted because its validity could not be proven in a subsequent institutional investigation [46]. However, a meta-analysis of smaller studies on patients with primary glomerulonephritis and proteinuria showed that double RAS inhibition was safe, and was more effective than single inhibition in treating proteinuria [47]. Moreover, patients treated according to the multimodal approach to proteinuria called 'remission clinic', which includes double RAS inhibition, had a strongly decreased risk of progression of renal disease compared with historic control patients on standard therapy titrated to BP, especially those with non-diabetic proteinuric nephropathies[48].

Concerns have been raised about double RAS inhibition because combined treatment of ramipril and telmisartan increased the risk of the combined primary renal outcome of death, serum creatinine doubling or dialysis compared with either agent alone in a large study comprising 25,620 patients with atherosclerotic disease and/or diabetes with end-organ damage [49]. However, it is important to realize that this increase in risk was mainly driven by the increased incidence of acute temporary hemodialysis, conceivably a treatment-related acute effect on renal hemodynamics that is reversible upon treatment withdrawal and does not indicate chronic renal disease progression. Moreover, 96% of patients in this study had normo- or microalbuminuria, and these results should not be generalized to patients with proteinuric nephropathies, in whom intensive proteinuria reduction will halt the vicious circle of progressive renal damage that is sustained by increasing levels of proteinuria [50].

## Renal functional improvement in humans treated with RAS inhibition

The studies mentioned above have predominantly used prevention of progression of albuminuria or deterioration of kidney function as the outcome of interest. Not many studies have addressed the possibility of long-term remission or even regression of proteinuric kidney disease with angiotensin II inhibition (Table 1). Preceded by several case reports [51,52], the first trial evidence that ACE inhibition may induce remission of nephrotic range proteinuria and stabilization of kidney function was provided by the Captopril Study, in which seven of 42 (16.7%) T1DM patients treated with captopril showed remission of nephrotic range proteinuria and stabilization of serum creatinine levels, compared with only one of 66 (1.5%) patients assigned to placebo [53]. After 1 year of follow-up, the single patient treated with placebo was also switched to ACE inhibition because of inadequate BP control. After nearly 8 years of follow-up, six of the seven patients for whom data were available remained in remission, whereas only one patient had progressed to ESRF [54]. Although better systolic BP control in the captopril group would have contributed to these findings, it was suggested that treatment with ACE inhibition can reverse the usually relentlessly progressive course of nephrotic range proteinuria in a subgroup of patients with T1DM.

**Table 1:** Definition of progression, remission and regression of proteinuric chronic nephropathies.

	Progression	Remission	Regression
Proteinuria, g/24 hours	≥ 1	0.3 to 1	<0.3

Glomerular filtration rate	*Declining	Stable	Increasing

Renal structural changes	Worsening	Stable	Improving

* Faster than physiological decline associated with aging (1 ml/min/1.73 m^2^/year).

**Figure 1:**
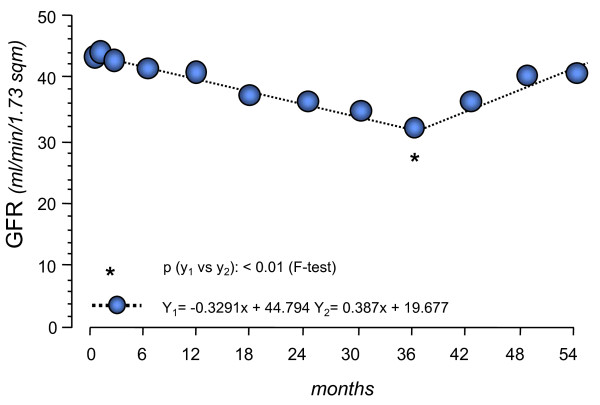
**GFR time-dependent changes over time in the 26 patients on prolonged ramipril therapy in the Ramipril Efficacy in Nephropathy (REIN) study**. The breakpoint analysis found that GFR time-dependent changes could not be described by one single first-degree equation, but rather by two different equations describing randomization to 36 months and from 36 to 54 months, respectively. Y_1 _and Y_2 _equations describe the interpolating curves before and after the breakpoint, respectively.

For non-diabetic patients, long-term follow-up of the REIN study showed that the rate of measured GFR decline progressively improved to a level of about 1 mL/min/1.73 m^2^/year after at least 5 years of continued ramipril use [55], which approximates the average age-related loss in GFR over time in healthy subjects [56]. Moreover, a breakpoint was identified in the slope of GFR changes over time, suggesting an improvement in mean GFR after 36 months of treatment. Indeed, 10 of 26 ramipril-treated patients had positive GFR slopes after the breakpoint. Patients with a positive GFR slope had a larger decrease in proteinuria compared with those in whom the slope improved but remained negative (Figure 1) [55]. Taken together, the findings of the Captopril and REIN studies, although not biopsy-proven, suggest that upon treatment with ACE inhibitors, recovery of kidney function due to kidney repair or regeneration is possible both in diabetic and in non-diabetic chronic proteinuric nephropathies.

## Renal structural improvement in animals treated with RAS inhibition

**Figure 2:**
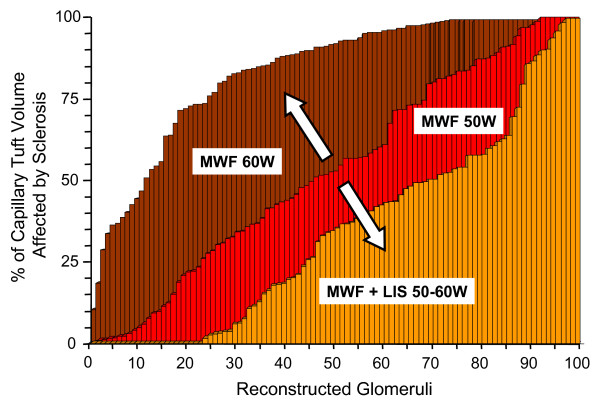
**Regression of glomerulosclerosis by ACE inhibition in an experimental model for progressive kidney disease**. Distribution of nonsclerosed capillary tissue as estimated by serial section reconstruction of the entire capillary tuft in Munich Wistar-Frömter rats at 50 weeks of age (in red), in untreated rats at 60 weeks of age, (in brown) and in animals treated with the ACE inhibitor lisinopril from 50-60 weeks of age (in yellow). In total, 100 glomeruli have been completely reconstructed in each animal group. Numbers in the abscissa represent reconstructed glomerular capillary tufts.

In a broad range of animal models of proteinuric disease, treatment with ACE inhibitors, ARBs, or both has been shown not only to prevent progressive renal damage, but also to induce regression of glomerulosclerotic, tubulointerstitial and vascular lesions [57-67]. Remuzzi *et al*. developed a technique for three-dimensional reconstruction of the glomerular capillary tuft, thus permitting a more accurate estimation of the extent of glomerulosclerosis, which is underestimated by currently employed two-dimensional imaging methods. Using this method, it was possible to show that in rats with advanced non-diabetic proteinuric nephropathy, administration of high-dose ACE inhibitors reduced the volume of sclerosis in most glomeruli (unless they were almost totally sclerosed), and increased the volume of normal capillary tissue by up to 40% (Figure 2)[66]. Other investigators have shown that the beneficial effects of angiotensin II inhibition on renal structural changes are dose-dependent and that the ideal dose may be higher than those recommended for antihypertensive treatment [59,60]. Moreover, a combined approach with ACE inhibitor, ARB and statin therapy was shown to be superior to combined treatment with ACE inhibitor and ARB, and led to complete regression of proteinuria and prevention of renal failure in a rat model of passive Heymann nephritis [68]. These studies suggest that maximization of antiproteinuric therapy is warranted to optimize the regeneration capacity of the kidney.

## Evidence for renal structural improvement with RAS inhibition in humans

Owing to the difficulties in obtaining repeat biopsies, only a few investigators have had the opportunity to study whether the human kidney is capable of repairing or regenerating chronically damaged renal tissue. The proof of concept that kidney repair is indeed possible in humans was provided by Fioretto *et al*., who studied biopsies of eight patients with T1DM and mild to advanced diabetic nephropathy after pancreas transplantation [69]. Five years after transplantation, no structural improvements were observed, but after 10 years, there was a significant decrease in glomerular and tubular basement membrane thickness and in mesangial fractional volume. Change in the latter was significantly correlated with the change in urinary albumin excretion rate. Kimmelstiel-Wilson nodules, typical of diabetic nephropathy, disappeared. Pancreas transplantation was accompanied by a significant decrease in creatinine clearance at 1 year of follow-up in all patients. This may have been a result of immunosuppressive treatment with ciclosporin; however, during the subsequent 9 years of follow-up, creatinine clearance remained remarkably stable and even improved in some patients, although it did not return to pre-transplantation values. These findings indicate that kidney repair is possible in diabetic nephropathy, but just as diabetic lesions take a long time to develop, they also take a long time to disappear.

In non-diabetic nephropathy also, kidney repair is possible. A study in proteinuric patients with idiopathic membranous nephropathy treated with the anti-CD20 monoclonal antibody rituximab added to the evidence in favor of kidney repair in humans [48]. In seven patients in whom complete remission of proteinuria had been obtained, biopsies were repeated after a median of 21 months after rituximab administration. The characteristic subepithelial electron-dense immune deposits were completely or partially reabsorbed, and staining for IgG4 was significantly decreased. Importantly, foot process effacement and loss of intact slit diaphragms were largely reversed. Inactivation by rituximab of the autoimmune pathways underlying idiopathic membranous nephropathy may thus allow restoration of the glomerular structure.

### Renal structural improvement with RAS inhibition in non-diabetic nephropathies

To our knowledge, no randomized studies are available investigating renal structural changes with angiotensin II inhibition in non-diabetic nephropathies. In an observational study, 15 patients with mild to moderate biopsy-proven IgA and non-IgA mesangial proliferative glomerulonephritis and relatively mild proteinuria were treated with an ARB for an average of 28 months. Although the global glomerular sclerosis ratio was not significantly altered by treatment with an ARB, 13 of the 15 patients showed a decrease in mesangial matrix expansion and interstitial fibrosis. Because treatment was associated with a significant decrease in proteinuria and systolic BP, no conclusion can be drawn from this study about a possible class-specific effect of ARBs [70].

### Renal structural improvement with RAS inhibition in diabetic nephropathy

Several studies have attempted to investigate the effect of angiotensin II inhibition on renal structural changes in humans. Small studies in microalbuminuric T1DM patients treated with enalapril, perindopril or the β-blocker metaprolol showed a decrease in glomerular basement membrane thickness after 3-4 years of follow-up[71,72]. Although a similar trend was present, this finding was not confirmed in a larger study in 59 T1DM microalbuminuric patients treated with enalapril or placebo[73]. In this study, however, mean glomerular and mesangial volumes were significantly increased in the placebo group compared with the enalapril group after 5 years of follow-up. A smaller study in 13 normoalbuminuric normotensive T1DM patients also showed a significant decrease in mesangial fractional volume after 5 years of treatment with the ARB candesartan. However, this decrease was accompanied by a decrease in BP, thus precluding a conclusion about a possible class-specific effect[74]. Recently, a large study on the renal and retinal effects of angiotensin II inhibition in 256 normoalbuminuric and normotensive patients with T1DM revealed that both ACE inhibition and ARB delayed the progression of retinopathy, but not nephropathy. Although sequential biopsies showed that the increase in the primary outcome of mesangial fractional volume after 5 years of follow-up was statistically significant in the placebo and ARB-treated groups, but not in the group treated with an ACE inhibitor, statistical comparisons between treatment groups revealed no significant differences [75]. Similarly, a study in 50 micro- and macroalbuminuric T1DM patients with preserved renal function but with histological evidence of diabetic nephropathy at baseline failed to detect any improvement in renal structural abnormalities after 3 years of treatment with enalapril or nifedipine compared with placebo [76]. Finally, for patients with T2DM, the effects of ACE inhibition on renal structural changes were investigated in 19 patients with micro- or macroalbuminuria who were randomized to treatment with perindopril or placebo [77]. After 2 years of follow-up, a significant increase in interstitial fractional volume was seen in the placebo group, which was absent in the perindopril-treated patients. The data also suggested that treatment with perindopril stabilized the percentage of sclerosed glomeruli in patients with T2DM, but this could not be confirmed in a smaller sample of 11 patients for whom electron microscopy data were available [78].

Although some of the studies cited above hint at the possibility of an effect of angiotensin II inhibition on renal structural changes in diabetic patients, they are inconsistent, and do not allow distinguishing between a specific effect of angiotensin II inhibition or of blood-pressure lowering in general. On the other hand, the negative results reported by other studies should not be taken as evidence for the absence of a beneficial effect of angiotensin II blockade, as some of these studies included patients at a very early stage of diabetic nephropathy or had only very small numbers. All studies had a relatively short duration of follow-up, and it should be kept in mind that, as described above, the effects of pancreas transplantation on renal structural changes in T1DM took 10 years to become evident [69].

## New therapeutic perspectives to optimize RAS inhibition

Blockade of the RAS at several sites has recently been proposed as a way to optimize antiproteinuric treatment in patients unresponsive to maximum combined ACE inhibitor and ARB therapy. Aldigier *et al*. showed that in remnant kidney rats the anti-aldosterone spironolactone promoted regression of glomerulosclerosis in 33% of cases when used alone, a percentage that increased further when nonspecific antihypertensive treatment or losartan were added [79]. Promising results in proteinuria reduction have also been obtained with aldosterone antagonists in patients unresponsive to single or dual RAS blockade [80]. Two recent meta-analyses found that add-on therapy with anti-aldosterone therapy reduces proteinuria in patients already on ACE inhibitor or ARB therapy, but this approach carries a high risk of hyperkalemia, especially in patients with advanced renal failure [81,82]. Moreover, response to anti-aldosterone therapy is not a uniform finding, especially in patients with non-diabetic nephropathies [83].

Renin is another component of the RAS that can be targeted. In the transgenic mRen-2 rat model, levels of plasma and tissue renin and other RAS components are altered, leading to hypertension. In mRen-2 rats with streptozotocin-induced diabetes, the direct renin inhibitor aliskiren reduced renal gene expression of TGF-β1 and collagen I, but not collagens III and IV, and also reduced expression of the (pro-)renin receptor in glomeruli, tubules and cortical vessels[84]. Other studies showed that aliskiren reduced albuminuria and glomerulosclerosis to a similar extent as the ACE inhibitor perindopril, despite lower BP reduction [85], and in non-diabetic mRen-2 rats, aliskiren and the ARB irbesartan similarly attenuated glomerular structural and functional changes[86]. In humans, aliskiren, in addition to losartan, was shown to significantly reduce the urinary albumin excretion rate compared with placebo in hypertensive patients with T2DM nephropathy[87]. However, it is not known whether this effect is superior to that which could have been obtained with upwardly titrated doses of losartan or with the combination of losartan with an ACE inhibitor. Given the high costs of this new drug, especially in comparison with ACE inhibitors, this issue needs to be resolved before aliskiren is routinely prescribed in everyday clinical practice.

Finally, growing evidence is accumulating of an antiproteinuric and nephroprotective effect of vitamin D, possibly acting again, at least in part, through RAS inhibition. Indeed, vitamin D receptor-null mice show increased levels of renin, whereas administration of vitamin D to wild-type animals suppresses renin mRNA levels in the kidney [88]. In remnant kidney rats, vitamin D administration significantly reduced glomerular hypertrophy over placebo [89]. A preliminary report from a randomized placebo-controlled clinical trial recently showed that 2 μg/day of paricalcitol added on to RAS inhibitor therapy decreased albuminuria by > 30% in a large cohort of patients with T2DM and overt nephropathy, whereas the smaller dose of 1 μg/day had a non-significant effect on urinary albumin excretion, similar to that of placebo [90].

## Mechanisms of renal disease regression

Two main, non-mutually exclusive mechanisms may be advocated to explain improved renal function and regression of histological lesions with angiotensin II inhibition. First, angiotensin II inhibition itself may favor kidney repair beyond reduction in BP and proteinuria, by affecting ECM deposition and podocyte structure and function. Alternatively, the kidney has an inherited capability to repair itself once the chronic insult (for example proteinuria) has been eliminated or attenuated. The evidence supporting these two hypotheses will be described below.

### Direct effects of angiotensin II inhibition

Several mechanisms have been proposed by which angiotensin II inhibition may directly promote regression of renal injury. A prominent feature of glomerulosclerosis is increased deposition of ECM, which is brought about by a change in the balance between breakdown proteinases and their inhibitors [7]. Results from animal studies have suggested several pathways by which angiotensin II may affect this balance. A key player in the deposition of ECM is transforming growth factor-β (TGF-β). In various rat models of progressive nephropathy, elevated TGF-β expression normalized upon treatment with an ACE inhibitor or ARB [58,66]. Conversely, TGF-β expression did not normalize upon treatment with hydralazine, suggesting an intrinsic effect of angiotensin II inhibition and not merely of BP reduction [58]. Importantly, repeated kidney biopsies in humans with T2DM and albuminuria after 2 years of treatment with the ACE inhibitor perindopril versus placebo showed decreased expression of the TGF-β1 gene and diminished downstream activation [91]. Another important factor is plasminogen activator inhibitor (PAI)-1, an inhibitor of matrix degradation with known profibrotic activity [92]. Its production can be directly induced by angiotensin II via the AT1 receptor [93]. After five-sixths nephrectomy in rats, regression of glomerulosclerotic lesions induced by treatment with ACE inhibitors and/or ARB was linked to decreased PAI-1 mRNA expression and reduced PAI-1 immunostaining on biopsy [61]. In the same study, mRNA expression of another inhibitor of proteolysis, tissue inhibitor of metalloprotease (TIMP)-1, was also reduced Finally, although increased production of matrix metalloproteinase (MMP)-2 and MMP-9 might favor regression as they promote ECM turnover and collagen breakdown, they were in fact reduced in the aforementioned study. In another study, however, increased MMP-2 and -9 levels returned to normal after 4 weeks of losartan treatment, concomitantly with normalization of collagen I and IV expression [58,61]. The role of MMPs in promoting regression of glomerular lesions therefore remains to be determined.

Several studies have suggested that angiotensin II has a direct effect on glomerular permselectivity [94,95]. In mice, altered organization of F-actin fibers and redistribution of the murine podocyte foot process protein zonula occludens-1 induced by angiotensin II was shown to be accompanied by increased protein permeability. Stabilization of F-actin prevented these changes [8]. Therefore, reorganization of the actin cytoskeleton, which is crucial to preserve podocyte-podocyte interactions, seems to be the underlying molecular mechanism of angiotensin II-induced glomerular permselective dysfunction, which may be inhibited by targeted treatment. Furthermore, angiotensin II receptor blockade was shown to reverse the decrease in vascular endothelial growth factor (VEGF)-A and angiopoietin-1 production by damaged podocytes [96]. These factors are all implicated in glomerular endothelial cell survival, and restoration of their production after glomerular injury may be important for capillary remodeling during regression of glomerulosclerotic lesions [57,66]. Finally, it was recently shown that lisinopril not only halted age-related podocyte loss in a rat model of spontaneous glomerular injury, but even increased the podocyte number compared with age-matched untreated animals [97]. These findings are in contrast to a previous report in which podocyte numbers were not affected by treatment with high-dose enalapril in five-sixths nephrectomized rats [57]. The use of different animal models is a possible explanation for these contradictory findings. Given that podocytes are highly differentiated cells, the finding of increased numbers is surprising. The authors of that report speculated that the limited proliferative capacity of podocytes is facilitated by ACE inhibition, or alternatively that parietal epithelial cells, which are increased with ACE inhibition, may serve as progenitor cells for podocytes and migrate from Bowman's capsule into the capillary tuft [97].

Finally, in cell cultures, proliferation of glomerular mesangial and endothelial cells is stimulated by angiotensin II [98]. Consistent with these findings, treatment with high-dose enalapril was associated with diminished mesangial cell proliferation and normalization of the number of endothelial cells, both of which are increased in untreated rats after subtotal nephrectomy [57]. However, from this study it cannot be concluded whether these effects can be directly attributed to angiotensin II inhibition or whether they are secondary responses to other components, such as decreased blood pressure or reduced glomerular scarring.

### Mechanisms of renal self-repair

Regression of glomerulosclerosis and neoformation of glomerular tissue has been linked to progenitor cells in the bone-marrow or inside the kidney [4,99-102]. Bone marrow cells act as a reservoir for glomerular mesangial cells in rodents [103], and cross-bone-marrow transplantation from young to old mice allows a partial regression of structural lesions associated with aging [101]. When injected after cisplatin injury, bone-marrow mesenchymal stem cells accelerate morphological and functional repair of the injured nephrons [104]. However, incorporation of mesenchymal cells into regenerating renal epithelium is a rare event, hence it has been hypothesized that their regenerative effect is most likely mediated by paracrine mechanisms related to the production of a broad array of mediators and growth factors with immunosuppressive, anti-inflammatory, antiapoptotic and proliferative effects [105]. Renal cell precursors also reside inside the kidney. Renal stem cells expressing the embryonic markers CD24 and CD133 have been identified in adult human kidney at the urinary pole of Bowman's capsule. Of note, the same markers identify common progenitors of podocytes and tubular cells during renal development [106]. Accordingly, experimental studies showed the presence of transitional cells exhibiting a mixed phenotype of parietal epithelial cells and podocytes at the vascular pole of the glomerulus [107]. Therefore, CD24+ CD133+ cells might be responsible for regeneration of both podocytes and tubular structures after injury, at least in mice [108,109]. In addition, evidence exists that endothelial and mesangial cells can proliferate in some circumstances, and that podocytes may even promote capillary growth by stimulating proliferation and migration of glomerular endothelial cells [102,110]. Moreover, it is well known that tubular epithelium has self-regenerating capacity after acute kidney injury, upon which differentiated tubular cells proliferate and migrate to replace damaged cells [111].

Stem cells have also been identified in proximal and distal tubuli and peritubular capillaries, although their role in and relative effect on kidney regeneration still has to be defined [112]. Finally, the renal papilla can also be a niche for kidney stem cells, and these cells were shown to start proliferating after renal ischemia [113]. Thus, various mechanisms have been proposed by which the kidney is able to repair itself, although the relative importance of each of these mechanisms remains to be elucidated [114].

## Conclusion

Different renal injuries eventually lead to reduced nephron numbers and increased intraglomerular pressure in the remaining nephrons. This results in increased urinary protein losses that, through a toxic effect on tubular cells, promote progressive renal scarring and function decline. Experimental studies and clinical trials clearly showed that strict BP control and maximal inhibition of RAS can reduce proteinuria and the rate of renal function decline. Notably, these therapies may halt and even promote regression of renal injury in some patients. Kidney repair induced by angiotensin II antagonists may either result from inhibition of the direct effects of angiotensin II on podocytes, glomerular permselectivity and fibrogenesis, or from the intrinsic capability of the kidney to repair itself once elements sustaining disease progression, such as hypertension and proteinuria, are controlled. A more in-depth understanding of the mechanisms by which RAS inhibition may retard or even reverse renal disease progression may significantly improve the morbidity and mortality associated with chronic kidney disease.

## Competing interests

The authors declare that they have no competing interests.

## Authors' contributions

All authors have been involved in the drafting of the manuscript and have revised it critically for important intellectual content. All authors read and approved the final manuscript.
